# Treatment of Placenta Increta With High-Intensity Focused Ultrasound Ablation and Leaving the Placenta *in situ*: A Multicenter Comparative Study

**DOI:** 10.3389/fmed.2022.871528

**Published:** 2022-04-07

**Authors:** Xiaoping Guan, Xiaoqin Huang, Min Ye, Guohua Huang, Xiao Xiao, Jinyun Chen

**Affiliations:** ^1^State Key Laboratory of Ultrasound in Medicine and Engineering, College of Biomedical Engineering, Chongqing Medical University, Chongqing, China; ^2^Chongqing Key Laboratory of Biomedical Engineering, Chongqing Medical University, Chongqing, China; ^3^Department of Gynecology, Neijiang First People’s Hospital, Neijiang, China; ^4^Department of Gynecology, Suining Central Hospital, Suining, China; ^5^Department of Gynecology, Chongqing Haifu Hospital, Chongqing, China

**Keywords:** placenta increta, high-intensity focused ultrasound (HIFU), focused ultrasound therapy, leaving the placenta *in situ*, placenta accreta spectrum, abnormally invasive placentation

## Abstract

**Objective:**

To explore the feasibility of simple high-intensity focused ultrasound (HIFU) ablation for placenta increta.

**Methods:**

Ninety-five patients after a vaginal delivery were enrolled in this retrospective cohort study, 53 patients were treated with simple HIFU ablation, and 42 patients were treated with HIFU followed by uterine curettage.

**Results:**

All 95 patients were successfully treated with a single-session HIFU procedure, and in the control group, the necrotic placental tissue was removed with curettage. Vaginal hemorrhage did not occur in either group. The duration of bloody lochia was 25.9 ± 8.6 days in the sHIFU group and 24.2 ± 8.8 days in the control group (*P* > 0.05). The median serum human chorionic gonadotropin (HCG) level was 3,222 mIU/mL and 2,838 mIU/mL in the sHIFU and control groups, respectively, which decreased and returned to normal within 30 days, and the differences were not significantly on comparing the blood HCG level in the two groups at 7, 15, and 30 days after HIFU (all *P* > 0.05). Decreased menstrual volume occurred in 85.71% of patients in the control group, which was higher than that in the sHIFU group (23.08%) (χ^2^ = 6.839, *P* < 0.001). During 2–8 years of follow-up, six pregnancies occurred in the sHIFU group without any recurrence of placenta increta, three pregnancies occurred in the control group, and one patient developed a repeat placenta increta.

**Conclusion:**

Simple HIFU treatment is safe and effective for postpartum placenta increta and leaving the placenta *in situ*. It is a promising option for patients who wish to preserve their fertility and conceive.

## Introduction

Placenta accreta spectrum (PAS) refers to the range of pathologic adherence of the placenta, including placenta increta, placenta percreta, and placenta accreta (1). Placenta increta is defined as invasion of the placenta into the myometrium, but not beyond (2). Abnormally invasive placentation (AIP) is observed clinically when the placenta cannot be separated from the uterus (3). The MRI features considered indicative of invasive placenta previa are as follows (4–6): (1) myometrial thinning: the myometrium thins and the typical trilaminar appearance is undetectable; (2) interrupted myometrium: the myometrium is interrupted abruptly at the site of focal placenta bulging; and (3) loss of the placental-myometrial interface: the thin hypointense layer between the placenta and myometrium disappears. When an abnormally invasive placenta develops, the placenta may not be completely separated from the uterus at the time of delivery, resulting in potentially life-threatening massive intrapartum or postpartum hemorrhage and associated morbidities, such as multisystem organ failure, disseminated intravascular coagulation, and even death (2).

Leaving the placenta *in situ* after delivery of the fetus has been utilized as a treatment option in multiple studies, but it is generally used in conjunction with other conservative modalities (2). The recent International Federation of Gynecology and Obstetrics (FIGO) consensus guidelines have recommended “leaving the placenta *in situ*” as a suitable option with close follow-up in hospitals with adequate expertise (7). Infection and hemorrhage and reoperative interventions are associated with high morbidity rates during observation and can even be life-threatening for the mother. Therefore, if the placental blood supply can be blocked in the clinical strategy of “leaving the placenta *in situ*,” it may be an effective idea to reduce the maternal morbidity. The 2018 FIGO placenta accreta disease guidelines clearly indicate the advantages of high-intensity focused ultrasound (HIFU) for the treatment of PAS (7).

High-intensity focused ultrasound is an *in vitro* low-intensity ultrasound focused on the target area *in vivo*, which forms a high-energy density focal point and causes rapid heating of the tissue in the focal area and coagulative necrosis of the target area within a short period of time, and thus, it achieves the purpose of treating the disease. At present, HIFU has been widely used in clinical practice to block the blood supply of uterine fibroids to induce coagulative necrosis. In a study including 12 patients with PAS after vaginal delivery treated with HIFU, one case could clear off the coagulated necrotic placental tissue and the remaining patients had a mean time to residual placental degeneration of 36.9 days. HIFU treatment did not increase the risk of infection or bleeding, and none of the patients required hysterectomy (8). However, there is still a lack of clinical evidence on whether placenta increta can be treated with HIFU ablation alone and leaving the placenta *in situ*. The aim of this study was to confirm the feasibility, safety, and efficacy of HIFU treatment alone in the treatment of placenta increta by a combined comparative study of HIFU treatment alone and HIFU treatment followed by curettage.

## Materials and Methods

### Patients

From 2013 to 2020, 95 women with postpartum placenta increta were treated in the First People’s Hospital of Neijiang City, Suining Central Hospital and Chongqing Haifu Hospital, and were enrolled in this retrospective cohort study. Inclusion criteria were as follows: (1) vaginal delivery, incomplete delivery of the placenta, and close adhesion of the placenta to the uterine muscle wall that could not be detached on palpation during freehand placenta removal. (2) Vital signs were stable and there were no signs of active hemorrhage. (3) The diagnosis of placenta increta was confirmed by MRI. (4) Intention to be treated with HIFU and signature on the written informed consent form. Exclusion criteria were as follows: (1) penetrating placenta increta; (2) occurrence of major bleeding (bleeding volume ≥500 mL); (3) residual placenta is large and extends to cervix or vagina; (4) current infection with body temperature ≥38.5°C. The study was approved by the ethics committee of the hospital (IRB approval number: 2020027). The Chinese Clinical Trial Registry (a non-profit organization, established according to both the WHO International Clinical Trials Register Platform Standard and the Ottawa Group Standard) provided full approval for the study protocol, recruitment materials, and consent form (Registration No. ChiCTR2200056055).^1^

### High-Intensity Focused Ultrasound Ablation

**FIGURE 1:**
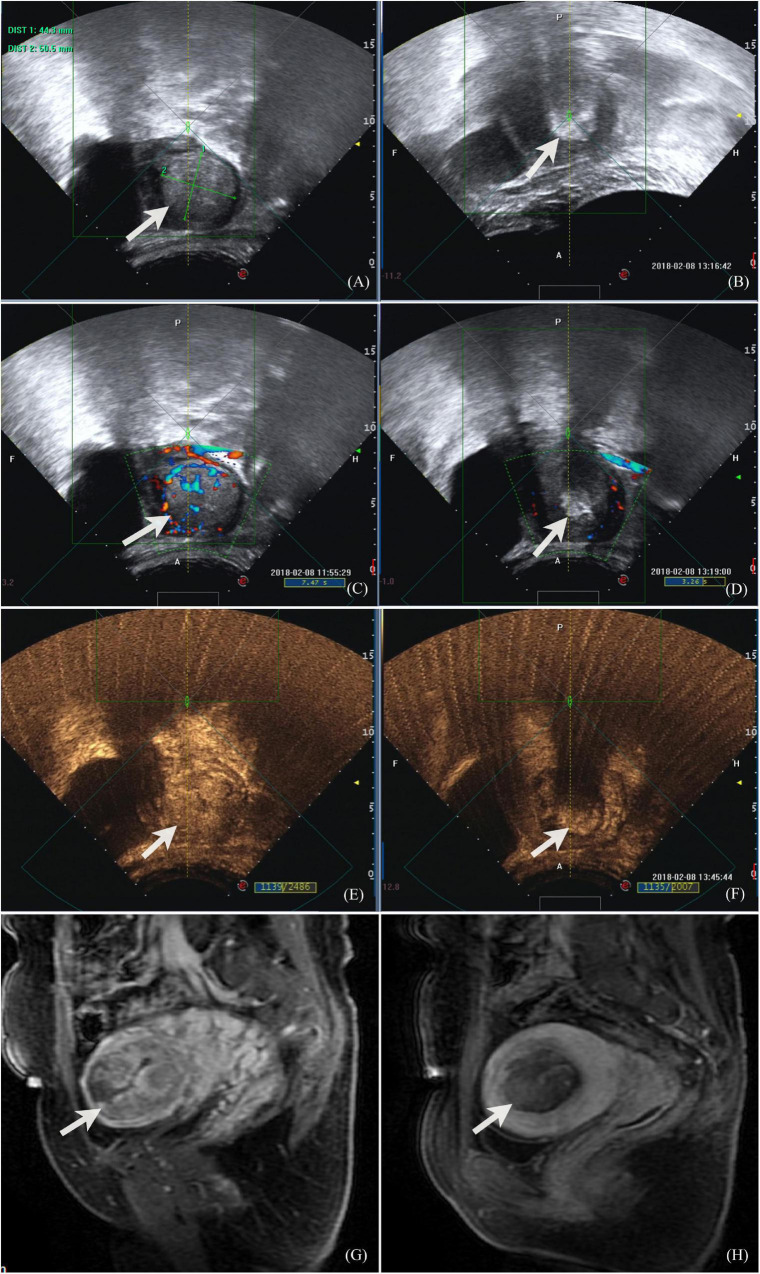
Ultrasound and MR images of a 32-year-old patient with placenta increta. **(A)** Before HIFU ablation, monitoring ultrasound showed placenta invasion on the fundus-posterior wall of the uterus, about 50.5 × 44.3 mm, with thin myometrium surrounding; **(B)** during the procedure of HIFU ablation, the monitoring images showed that the target area was clumped with enhanced echo; **(C)** color Doppler flow imaging showed abundant blood flow signals both in the lesion and surrounding before HIFU ablation; **(D)** color Doppler flow imaging showed that the grayscale increased in the lesion and no blood flow signal was observed, also peripheral blood flow signal decreased and disappeared after HIFU ablation; **(E)** contrast-enhanced ultrasound showed that there was abundant blood perfusion in the lesion before HIFU ablation, and there were lacunar areas with less blood flow; **(F)** contrast-enhanced ultrasound showed that after HIFU ablation, the lesions were covered by grayscale increased images, and the posterior acoustic attenuation was observed. There was no blood perfusion in the lesions, and the boundary between the lesions and the surrounding uterine myometrium was clear; **(G)** contrast enhanced MR image showed that abnormal signal lesion enhanced in the intrauterine before HIFU and thin myometrium in the fundus of uterus; **(H)** contrast enhanced MR image 1 day after HIFU showed that the lesion in the uterine cavity was ablated with no blood perfusion.

Ultrasound-guided HIFU was performed using a Focused Ultrasound Tumor Therapeutic System (Model-JC200, Chongqing Haifu Medical Technology Co., Ltd., Chongqing, China). The procedure of HIFU ablation has also been described in a previous publication (7). In summary, all patients were asked to consume only liquid food for 1 day, followed by a 6-h fasting period. An enema was performed on the morning of the treatment day. The hair on the abdominal wall from the umbilicus to the level of the upper margin of the pubic symphysis was shaved and degreased using 75% ethanol solution. The patient was positioned prone on the treatment table of the HIFU system with the abdominal wall in contact with degassed water over the transducer. The treatment plan was created automatically under ultrasonography guidance: the residual placenta increta was divided into slices of a thickness of 5 mm, acoustic power of 350–400 W was used, and HIFU treatment was terminated when guided ultrasonography showed a grayscale increase change in the target tissue or the signal of blood flow to the placental tissues disappeared. Contrast-enhanced ultrasound was also used to evaluate blood perfusion in the placental tissues (Supplementary Material). Patients were given low doses of fentanyl citrate injection (first 1 μg/kg) and midazolam (first 0.03 mg/kg, total combined dose ≤0.15 mg/kg) *via* the intravenous (IV) route during the procedure. The patient’s response was observed while the sonication was being performed, so that the patient was at the sedation level 3–4 Ramsay scale. Non-perfused Volume rate (NPVR) was measured on enhanced MRI after HIFU treatment. Monitoring and evaluation images are shown in Figure 1.

### Management After High-Intensity Focused Ultrasound

After HIFU treatment, patients were informed about two management options. Allowing the placenta to be kept *in situ* to wait for absorption and expulsion, or curettage with removal of necrotic lesions after HIFU ablation and possible reduction of the postoperative observation time, but there was a risk of uterine perforation and hemorrhage. Patients who voluntarily chose to undergo curettage served as the control group. Curettage was performed by a team of experienced gynecologists from each hospital under ultrasound imaging guidance within 5 days after the HIFU procedure.

### Follow-Up

Serum human chorionic gonadotropin (HCG) levels were rechecked on day 7, day 15, and day 30 after HIFU until return of normal levels. The time and volume of vaginal bleeding, the time and menstrual volume, and pregnancy achievement were assessed by a telephone interview.

### Statistical Methods

The statistical software SPSS 26.0 was used for statistical description. The categorical variables were expressed as frequencies and percentages, and continuous variables were expressed as medians (interquartile range, IQR). The intergroup comparison of the count data was performed by the Chi-square test or Fisher’s exact probability method. The Mann–Whitney U test was used for comparison of the measurement data between groups. Generalized estimating equations were used for intergroup comparisons of repeated measures at a test level of 0.05. Differences were considered statistically significant at *P* < 0.05.

## Results

Ninety-five patients were enrolled in this study. There were 53 patients in the sHIFU group; the median age was 29.0 years (IQR: 24.0–32.0 years); 12 women were primipara, 41 had a history of delivery and 21 cases underwent cesarean section; 32 females delivered at 28 weeks or more; the implanted placenta size (longest diameter) was 6.0 cm (IQR: 4.0–7.0 cm) and the area was 15.1 cm^2^ (IQR: 9.6–26.3 cm^2^) (longest diameter × vertical diameter of the longest diameter), and the placenta increta depth was 4.0 cm (IQR: 2.6–4.5 cm). In the control group, there were 42 patients, the median age was 28.0 years (IQR: 25.0–32.0 years); 5 women were primipara and 37 were multiparas, including 23 cases that underwent cesarean section; 18 females delivered at 28 weeks or more; the implanted placenta size was 7.0 cm (IQR: 5.2–9.0 cm) and the area was 31.3 cm^2^ (IQR: 15.8–51.8 cm^2^), and the placenta increta depth was 4.5 cm (IQR: 3.6–4.9 cm). Details are presented in Table 1.

**TABLE 1:** Comparison of clinical characteristics of patients in two groups.

	sHIFU group (*n* = 53)	Control group (*n* = 42)	*P*-value
Age (years)*	29.0 (24.0–32.0)	28.0 (25.0–32.0)	0.916
Number of gravidities*	3.0 (3.0–5.0)	3.0 (2.0–5.0)	0.753
Number of parturitions*	1.0 (1.0–2.0)	1.0 (1.0–2.0)	0.641
History of cesarean section (*n*, %)	21 (39.6%)	23 (54.8%)	0.154
**Gestational week (*n*, %)**			0.102
<28 Weeks	21 (39.6%)	24 (57.1%)	
≥28 Weeks	32 (60.4%)	18 (42.9%)	
Placenta increta size^1^ (cm)*	6.0 (4.0–7.0)	7.0 (5.2–9.0)	0.042
Placenta increta area^2^ (cm^2^)*	15.1 (9.6–26.3)	31.3 (15.8–51.8)	0.001
Placenta increta depth (cm)*	4.0 (2.6–4.5)	4.5 (3.6–4.9)	0.003
Serum HCG (mIU/mL)*	2,550.0 (996.5–5,523.0)	1,895.2 (217.6–5,321.0)	0.152
Sonication time (sec)*	660.0 (538.0–890.0)	710.0 (585.0–1,024.0)	0.438
Power (W)*	398.0 (394.0–400.0)	400.0 (396.0–400.0)	0.068
NPVR (%)*	83.0 (75.0–87.0)	81.0 (74.8–88.3)	0.922
Duration of bloody lochia (days)*	24.0 (19.0–31.0)	25.0 (16.0–31.0)	0.422
Tissue expulsion (days)*	9.0 (5.0–14.0)	3.0 (3.0–6.0)	<0.001
Menstrual resumption (days)*	62.0 (54.0–72.0)	70.0 (62.0–82.0)	0.007
Treatment time (min)*	71.0 (60.0–90.0)	66.0 (55.0–95.0)	0.860

*HCG, human chorionic gonadotropin; NPVR, non-perfused volume ratio.*

*Data are expressed as median (interquartile range).

*^1^The longest diameter of the implanted placenta.*

*^2^The area of the implanted placenta = 1/4 π multiplied by the longest diameter times the vertical diameter of the longest diameter.*

### Results of High-Intensity Focused Ultrasound Treatment

**FIGURE 2:**
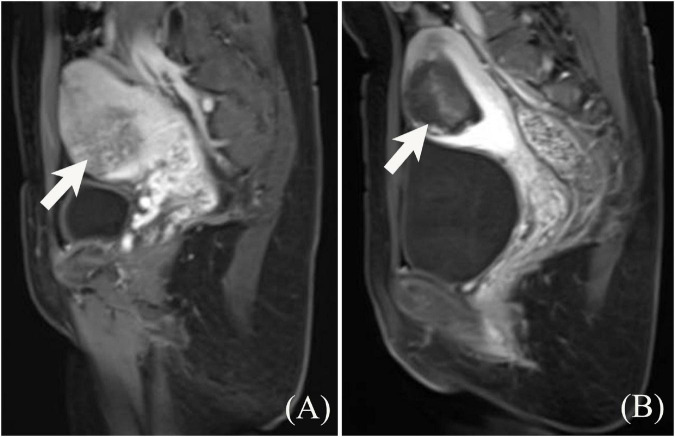
Contrast enhanced MRI of A 23-year-old woman with placenta increta before and after HIFU treatment. **(A)** The depth of placenta invasion was 3.7 cm, close to the uterine serous surface, and uniform enhancement of the implanted placenta with perfusion slightly below the myometrium. **(B)** After HIFU treatment, contrast enhanced MRI shows no perfusion of the placental tissue.

All 53 patients in the sHIFU group and 42 patients in the control group completed HIFU treatment as per the treatment plan, and all patients appeared to have a non-perfused area within the implanted placenta, as shown in Figure 2. Patients complained of discomfort, such as pain and abdominal distension in the sacrococcygeal region and treatment area during HIFU treatment, which was tolerable. No complications, such as skin burns and lower limb paralysis, occurred after treatment. The median power of HIFU treatment was 400.0 W (IQR: 395.0–400.0 W), treatment time was 70.0 min (IQR: 59.0–92.2 min), and sonication time was 700.0 s (IQR: 550.0–950.0 s). The median NPVR of the implanted placenta was 83.0% (IQR: 75.0–87.0%) in the sHIFU group and 81.0% (IQR: 74.8–88.3%) in the control group, and the difference between the two groups was not significant (*Z* = 0.098, *P* = 0.922).

### Tissue Expulsion and Vaginal Bleeding After Treatment

Forty-two patients were treated with curettage 1–5 days after HIFU, and necrotic tissue was expelled in 100% of the cases without any complications, such as uterine perforation. All 53 cases in the sHIFU group discharged necrotic tissues, while 41 cases in the control group experienced intermittent discharge of necrotic tissues after the curettage procedure. None of these 95 patients developed heavy bleeding after treatment, and only a few cases have a small volume of old blood vaginal discharge. The duration of bloody lochia was 24.0 days (IQR: 19.0–31.0 days) in the sHIFU group and 25.0 days (IQR: 16.0–31.0 days) in the control group, with no significant difference between the two groups (*Z* = 0.803, *P* = 0.422).

### Changes in Serum Human Chorionic Gonadotropin After Treatment

Serum HCG levels decreased sharply after treatment in both groups, and serum HCG levels in both groups returned to normal within 30 days after treatment. There was no statistically significant difference between the HCG level in the sHIFU group and the control group at 7, 15, and 30 days postoperatively (*P* > 0.05), as listed in Table 2. The “interaction of grouping factors and time” was also not statistically significant (*P* > 0.05); i.e., there was no difference in the time trend of serum HCG between the two groups, as shown in Table 2.

**TABLE 2:** Comparison of the serum HCG level changes before and after treatment (mIU/mL).

	Before treatment	After treatment		
		7 days	15 days	30 days
sHIFU group	2,550.0 (996.5–5,523.0)	288.0 (100.0–633.0)	78.0 (46.0–298.0)	<1.20 (<1.20, <1.20)
Control group	1,895.2 (217.6–5,321.0)	191.9 (44.8,- 5.5)	79.5 (22.5–214.6)	<1.20 (<1.20, <1.20)
*Z*-value	−1.432	−1.544	−1.225	−1.000
*P*-value	0.152	0.122	0.182	1.000

*Data are expressed as median (interquartile range).*

### Menstrual Resumption and Pregnancy Achievement

After HIFU treatment, menstruation resumed within 3 months in all 95 patients, and it took 62.0 days (IQR: 54.0–72.0 days) in the sHIFU group and 70.0 days (IQR: 6,254.0–82.0 days) in the control group; there was a significant difference between the two groups (*Z* = 2.685, *P* = 0.007). A total of 85.7% of patients in the control group experienced reduced menstrual volume, which was significantly higher than that in the sHIFU group (23.1%), with a significant difference (χ^2^ = 6.839, *P* = 0.000).

During 2–8 years of follow-up, six pregnancies in the sHIFU group and three pregnancies in the control group were delivered at full term, and eight cases experienced uneventful delivery of the placenta after delivery. In the sHIFU group, five cases delivered *via* cesarean section and one case underwent a vaginal delivery. In the control group, three cases delivered *via* cesarean section, and in one case, the placenta was incompletely detached and partially implanted for about 3 × 3 cm, bleeding on the detached surface was active with suturing, and then the bleeding was successfully stopped.

## Discussion

Placenta accreta occurs in approximately 1:1,000 deliveries with a reported range from 0.04% rising up to 0.9% (9, 10). According to a large population-based pregnancy cohort study, the incidence of PAS reach up to 2.1% (11). Placenta increta accounted for 29.8% of PAS patients undergoing surgical management (12). Currently, there are clinical differences in the management of placenta increta (7, 13). For placenta increta combined with uncontrollable hemorrhage that endangers maternal life, hysterectomy remains the primary treatment (14). Hysterectomy deprives the patients of their fertility and normal menstrual cycles, and some studies have suggested that hysterectomy leads to premature ovarian failure and early onset of perimenopausal symptoms in women. “Leaving the placenta *in situ*” can successfully preserve the uterus and avoid hysterectomy in the majority of patients without any major bleeding, but there is still a risk of infection and bleeding, and whether residual placenta mechanization will affect later pregnancy is still a clinical concern. Focused ultrasound therapy is a non-invasive treatment technique that has been widely used in the clinical treatment of many diseases in recent years, and it has achieved satisfactory clinical outcomes (15–19). The HIFU ablation technique is used for the treatment of placenta increta, which is based on the strategy of “leaving the placenta *in situ*,” where HIFU ablation causes *in situ* necrosis of the residual placental tissue, vascular blockage, peeling off of the necrotic tissue, and reduction in the number of medical operations, such as curettage. HIFU alone is feasible option for the treatment of patients who develop non-major bleeding postpartum placenta increta with *in situ* leaving of the placenta, and the return of menstruation and pregnancy achievement in some patients after treatment reveal its unique advantages.

The studies reported that HIFU combined with hysteroscopic resection is an effective and safe method for the management of placenta accreta (20, 21). But less is more. HIFU alone is more beneficial 53 patients were included in this study for HIFU ablation alone and 42 patients were treated with HIFU ablation followed by curettage; no significant differences were observed between the two groups in terms of patient age, gestational week and serum HCG. After HIFU ablation, the implanted placental lesion was necrotic *in situ*, and the lesion was not enhancing, as assessed by an enhanced MRI, leaving the placenta *in situ*, and avoiding the risk of re-injury of the uterus during curettage procedure. Disruption of the integrity of the uterine endometrium and smooth muscle layers of the myometrium is the main cause of placental invasion (22). Therefore, reducing intrauterine surgery is an important measure to avoid the occurrence of placental implantation in second pregnancy.

In this study, although the placental size and placental implantation depth were statistically greater in the control group than in the sHIFU group (*P* < 0.05), all HIFU ablations achieved technical success with a median NPVR ≥80%. The key to the clinical benefit of the HIFU ablation technique is to obtain a high NPVR, with target tissue structure and blood supply being the major influencing factors. Keserci et al. (23) reported that enhanced MRI evaluation of adenomyosis with blood perfusion intensity below the myometrium obtained a mean NPVR of 89.2%, which was significantly higher than the NPVR of 42.9% in the group with blood perfusion equal to that above the myometrium. The placenta is an organ for material exchange between the mother and the fetus. There are two sets of blood circulation in the placenta, maternal and fetal blood circulation. When a fetus is delivered, the maternal-fetal material exchange is discontinued, and the placenta is detached from the mother. When the implanted placenta cannot be detached, the placental decidua basalis can still obtain blood supply from the myometrium, but most of the blood vessels in the placenta are occluded and some tissues become necrotic and degenerated; therefore, most of the residual placenta can be easily ablated and a high NPVR can be obtained. It needs to be further investigated whether the effect of ablation can be predicted by imaging means, such as enhanced MRI.

Although the safety of HIFU for uterine fibroids and adenomyosis has been extensively studied and reviewed (24–26), its safety still needs to be considered in terms of case selection, preoperative preparation, intraoperative monitoring, and dose control (27). Firstly, placental implantation is very deep and there is a risk of damage to the ectopic bowel during HIFU ablation; hence, patients with placenta penetration were excluded from case selection in this study. Specific treatment strategies need to be considered for placenta penetration.

Secondly, near-field acoustic channel scarring is also an important factor affecting the safety assessment of HIFU ablation (27). Ultrasound-guided HIFU ablation systems, with cryogenic circulating degassed water as the coupling medium and a continuous cooling effect on the acoustic channel skin, have a high safety profile in patients with abdominal surgery-related scarring (28). Some scholars have used scar patches in MRgHIFU treatment to avoid damage to the scar when high NPVR is obtained (29). Due to different imaging principles, it needs to be further studied whether scar patches are suitable for USgHIFU. Thirdly, for placental implantation, the susceptibility of residual placenta to infection is a matter of concern and should therefore be taken into account while screening cases, and further consultation is needed in case of abnormalities by monitoring the temperature. Fulminant exacerbation of infection due to HIFU ablation should be avoided since it may be life-threatening for the patient. Finally, death of patients reported in the literature, although rare, raises a higher level of warning for the clinical application of HIFU technology (27). In patients with placental implantation, the physiological situation is significantly different from that in non-pregnant women, and it is necessary to determine whether there are any problems related to vascular injury. In this early stage of HIFU technology application, attention needs to be paid to the possibility of uncertain risks in cases with pathological pregnancies, such as gestational hypertension disorder. Future studies should evaluate the safety of this technique in the context of obstetric conditions.

During the observation period, major bleeding did not occur in patients who underwent clearing and non-clearing of the uterus, and no obstetric complications, such as Asherman syndrome, were observed. A small amount of necrotic tissue was discharged vaginally, accompanied by only a small volume of old blood discharge. There were no statistically significant differences in the time to vaginal bloody lochia, time to HCG decline, and time for conversion to negative (*P* > 0.05). Therefore, leaving the placenta *in situ* after HIFU for treating postpartum placenta increta is a feasible and safe option, which has comparable efficacy to HIFU combined with curettage.

Individualized management is performed in young women with fertility preservation requirements (30). Menstrual recovery is an important outcome. In this study, patients had menstrual cycle resumption within 3 months after HIFU ablation leaving the placenta *in situ* and had a significantly lower incidence of menstrual volume decreased than in the HIFU combined with curettage group. Further study is needed to assess whether curettage induced the injury to the endometrium or affects the endometrial microenvironment. There is a lack of histological evidence on how the myometrium recovers in patients treated with leaving the placenta *in situ* after HIFU with the necrotic placental tissue expels spontaneously. However, on a long-term follow-up, nine women achieved a second pregnancy; eight women had an uneventful delivery of the placenta without any recurrence of placenta increta, and one case developed partial placenta increta along with the second pregnancy, and the uterus could still be preserved after intraoperative management by cesarean section. This finding suggests that the structure and physiological function of the myometrium and endometrium can be restored after HIFU ablation.

However, the present study has some limitations. The first limitation is the failure to establish the pathological diagnosis of implanted placenta and histological outcome evidence of myometrial and endometrial repair. Secondly, the small number of cases and design of the retrospective study may have resulted in patient selection biases, such as statistical differences in placenta size and depth of placental implantation; hence, the impact of patient selection on clinical application still needs to be considered, and they necessitate further validation of its clinical application in a prospective randomized controlled study. Further randomized controlled studies are needed to confirm whether the single-session HIFU treatment strategy of placental leaving *in situ* is superior to leaving the placenta *in situ* without any intervention in terms of the conception rate, pregnancy safety, and placenta accreta during postoperative pregnancy achievement, and whether this treatment strategy is superior to HIFU combined with curettage. In addition, patients who developed any major bleeding were excluded from this study, and it needs to be further explored whether effective hemostasis can be achieved by using the HIFU technique.

## Conclusion

In summary, single-session HIFU is safe and effective in treating postpartum placenta increta *in situ*. HIFU alone opens a new era of leaving the placenta *in situ* treatment of placenta increta and is a promising option for those who wish to preserve their fertility and experience pregnancy.

## Data Availability Statement

The original contributions presented in the study are included in the article/Supplementary Material, further inquiries can be directed to the corresponding author.

## Ethics Statement

The studies involving human participants were reviewed and approved by the Neijiang First People’s Hospital Clinical Research Ethics Committee. The patients/participants provided their written informed consent to participate in this study.

## Author Contributions

XG and JC contributed to the conception and design of the study, performed the statistical analysis, and wrote and revised the manuscript. XG, XH, MY, GH, XX, and JC were responsible for data acquisition and interpretation. All authors read and approved the final manuscript.

## Conflict of Interest

The authors declare that the research was conducted in the absence of any commercial or financial relationships that could be construed as a potential conflict of interest.

## Publisher’s Note

All claims expressed in this article are solely those of the authors and do not necessarily represent those of their affiliated organizations, or those of the publisher, the editors and the reviewers. Any product that may be evaluated in this article, or claim that may be made by its manufacturer, is not guaranteed or endorsed by the publisher.

## Footnotes

1
www.chictr.org.cn
